# Predictors and Trend in Attendance for Breast Cancer Screening in Lithuania, 2006–2014

**DOI:** 10.3390/ijerph16224535

**Published:** 2019-11-16

**Authors:** Vilma Kriaucioniene, Janina Petkeviciene

**Affiliations:** Faculty of Public Health, Academy of Medicine, Lithuanian University of Health Sciences, LT47181 Kaunas, Lithuania; janina.petkeviciene@lsmuni.lt

**Keywords:** breast cancer, screening, trend, attendance, predictors

## Abstract

In Lithuania, a Nationwide Breast Cancer (BC) Screening Program was launched in 2005, offering mammography for women aged 50 to 69 years, every other year. This study aimed to determine the trend in the attendance for mammography screening during 2006–2014 and to identify the factors that are predictive for participation in it. The study sample consisted of 1941 women aged 50–64 years, who participated in five cross-sectional biennial postal surveys of Lithuanian Health Behavior Monitoring, carried out in independent national random samples. The attendance for screening was identified if women reported having had a mammogram within the last two years. The proportion of women attending the screening was continuously increasing from 20.0% in 2006 up to 65.8% in 2014. The attendance for BC screening was associated with the participation in cervical cancer screening. A higher level of education, living in a city, frequent contact with a doctor, and healthy behaviors (fresh-vegetable consumption, physical activity, and absence of alcohol abuse) were associated with higher participation rates in BC screening. To increase BC screening uptake and to reduce inequalities in attendance, new strategies of organized BC screening program using systematic personal invitations are required in Lithuania.

## 1. Introduction

Breast cancer (BC) is the most frequently diagnosed female cancer around the world, accounting for almost one in four cancer cases in women [1]. In 2018, BC was the most common cancer sites (28.2% of all cancers) and the leading cause of death (16.2% of cancer death) in the European female population [2,3]. The highest incidence rates of BC were in Western and Northern Europe. In Lithuania, the estimated incidence rate was below the European average (80.6 vs. 100.9 per 100,000 women), while the mortality rate was higher than the European average (22.7 vs. 21.8 per 100,000 women), suggesting unfavorable survival [2].

Many studies have shown that well-organized cancer screening could be effective in reducing BC mortality [4,5,6,7]. When estimating the decline in BC mortality attributable to screening, it is very important to distinguish the screening effect from other effects, such as improvement in treatment or reduced use of hormone replacement therapy. According to the recent studies carried out in Norway, Denmark, and Ireland, mammography screening was associated with an additional benefit in reducing BC mortality above the benefits from improved treatment alone [8,9,10]. Women who participated in organized BC screening in Sweden had a 60% lower risk of dying from BC within 10 years after diagnosis compared with nonparticipants [11]. BC screening programs play an important role in the early diagnosis of cancer and enable women to have more choices of advanced treatment for early stage BC, increasing survival [11,12].

Following the European Union Council Recommendation of 2 December 2003, most European countries implemented organized, population-based breast cancer screening programs based on mammography [13,14,15]. Although opportunistic screening coexists in many countries, organized programs, which use central registers for invitation, monitoring, and evaluation and ensure quality control, are more likely to reduce BC mortality than opportunistic programs [16,17].

The effectiveness of a BC screening program is related to the coverage of the target population. In most developed countries, access to mammography screening is free of charge; however, inequalities in the attendance were identified in population-based studies from different geographical regions. Lower education, living in rural areas, and lower socioeconomic status were associated with lower uptake of mammography screening [16,18,19,20]. Previous studies carried out in Spain, Italy, and France showed that the implementation of an organized population-based screening program can diminish socioeconomic inequalities in access to screening [21,22,23]. Only a few studies analyzed the associations of health behavior and health factors with participation in BC screening, showing that women with an unhealthy lifestyle participated less often [18,24,25]. Data from the French National Program showed that being a heavy smoker significantly increased the likelihood of not being screened (OR = 1.84; 95% CI 1.44–2.37) [24]. Meanwhile, more frequent visits to a doctor, a higher number of chronic diseases, and poorer subjective health were associated with higher attendance for BC screening [24,25]. A study carried out in Portugal found that women having had at least one routine doctor appointment in the previous three months were nine-fold less likely to have never undergone screening mammography [24].

In Lithuania, a Nationwide Breast Cancer Screening Program was launched in 2005, offering mammography for women aged 50 to 69 years, every other year [15,26]. The program is financed by the National Health Insurance Fund, which is an institution of the National Health Insurance System paying for the health-care services provided to patients. There is no systematic personal invitation system for the whole target population. The mammography test can be offered during contact between a woman and a general practitioner in primary health-care centers. Thus, BC screening in Lithuania is more opportunistic than organized and does not assure adequate coverage. So far, very little data exist about associations of sociodemographic and lifestyle factors, as well as the usage of health services with participation in screening. To our knowledge, no study analyzed the longitudinal time trends and predictors of attendance for mammography screening in Lithuania. The information from this study is important for identifying, monitoring, and addressing any possible disparities in mammography coverage and may be useful for screening organizers to develop new invitation strategies and methods for women who have low willingness to participate in the screening program.

This study aimed to assess the trend in the attendance for mammography screening in Lithuania during 2006–2014 and to identify the sociodemographic, lifestyle and health-care service use factors that are predictive for participation in BC screening.

## 2. Materials and Methods

The data from five cross-sectional surveys of Lithuanian Health Behavior Monitoring carried out every second year throughout 2006–2014 were analyzed [27]. This study provides information on the trends in health behaviors and the usage of health services in the adult population. For every survey, a nationally representative simple random sample aged 20–64 years was drawn up from the National Population Register. The questionnaires with one reminder were sent to selected individuals. The Lithuanian Bioethics Committee approved all surveys (protocol number: 6B-10-61). Written informed consent for participation was obtained from all respondents.

The data of women aged 50–64 years (the target age group for the Nationwide Breast Cancer Screening Program) with available information on participation in BC screening (*n* = 1941) were analyzed in this study. Response rates for women of this age group were 60% in 2006, 63% in 2008, 58% in 2010, 51% in 2012, and 50% in 2014. Information on BC screening attendance was obtained from the questions: ‘Have you ever had a mammogram?’ with possible answer choices (1) yes, (2) no, and (3) I do not know; and ‘When you had the last mammogram?’ with possible answer choices (1) during the previous year, (2) one–two years ago, and (3) more than two years ago.

In Lithuania, the Nationwide Cervical Cancer Screening Program targeting all women aged 25–60 years and offering a conventional Pap smear test within a three-year interval started in 2004. To identify the association between participation in BC and cervical cancer screening, women were asked: ‘Have you ever had a Pap smear test for cervical cancer screening?’ Possible answer choices were as follows: (1) during the last 12 months; (2) one–three years ago; (3) more than three years ago; and (4) never.

The socio-demographic variables used in the analysis were education, place of residence, marital status, and ethnicity (Table 1). Women were divided into three groups according to the highest level of completed education: low education (primary education, incomplete secondary education, or secondary school), intermediate education (vocational school), and high education (college or university). According to the administrative classification of the residence, the respondents were categorized as living in cities (capital city Vilnius and four largest cities of Lithuania: Kaunas, Klaipėda, Šiauliai, and Panevėžys), towns (centers of municipalities and other towns with at least 2000 inhabitants), and villages. Marital status was dichotomized as ‘married’ and ‘unmarried’ (single, divorced, or widowed). Data on self-reported ethnic identity were categorized into Lithuanians and others (Russians, Poles, Belarussians, and all other ethnicities).

Women were divided into three groups by the number of visits to a doctor during the last year: no visit, 1–2 visits, and 3 or more visits. Analyzed health behaviors were smoking, consumption of strong alcoholic drinks (hard liquor), leisure-time physical activity, and fresh-vegetable consumption. Respondents were classified into the following smoking status groups: current daily smokers and others, which include occasional smokers, quitters, and never-smokers. The frequency of strong-alcoholic-drink consumption was dichotomized as ‘at least once a week’ and ‘less often or never’. Association of consumption of other types of alcohol, such as wine and beer, with attendance for BC screening was also analyzed, but only consumption of strong alcoholic drinks was a significant predictor of participation in screening and hence was included in the logistic regression model. Self-reported moderate-intensity leisure-time physical activity lasting at least half an hour was categorized into two groups: on two or more days a week and less often. According to the frequency of fresh-vegetable consumption, women were grouped in daily consumers and those consuming less often or never.

The analysis was performed by using the statistical package IBM SPSS Statistic 20 (IBM, Armonk, NY, USA). The categorical variables were presented as proportions. The normal approximation was used in the calculation of 95% confidence intervals for proportions. A χ^2^ test and Z-test with Bonferroni correction were used for comparisons of proportions. Secular trends in attendance and nonattendance for BC screening between 2006 and 2014 were examined by using linear regression analysis. The associations between attendance for mammography screening and sociodemographic, as well as health behavior, variables were evaluated by using logistic regression analysis. The *p*-values of less than 0.05 were considered statistically significant.

## 3. Results

**Figure 1:**
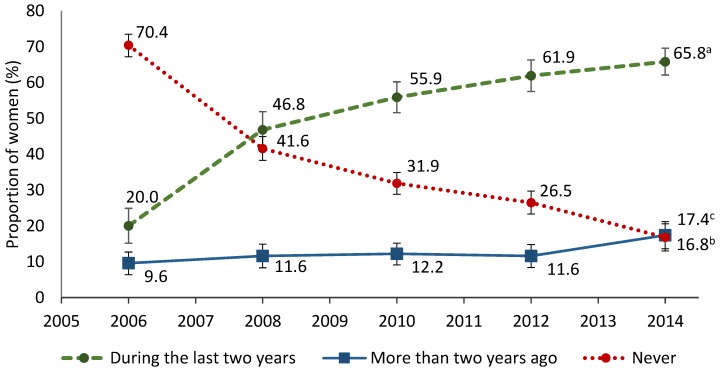
Trends in attendance and nonattendance for mammography screening in 2006–2014. Linear trend: ^a^ β = 10.7, *p* = 0.025; ^b^ β = −12.1, *p* = 0.017; ^c^ β = 1.4, *p* = 0.066.

The results regarding the trends in BC screening between 2006 and 2014 are displayed in Figure 1. Since 2006, the proportion of women reported having had a mammogram within the last two years was continuously increasing from 20% in 2006 up to 65.8% in 2014. The data of linear regression analysis showed a significant increase in this proportion of 10.7% per each two-year study period (*p* = 0.025). Although 70.4% of women reported never receiving a mammogram in 2006, this rate decreased significantly to 16.8% in 2014 (the decrease of 12.1% per each two-year study period, *p* = 0.017). During the study period, the proportion of women who reported having had a mammogram more than two years ago almost did not change.

**Figure 2:**
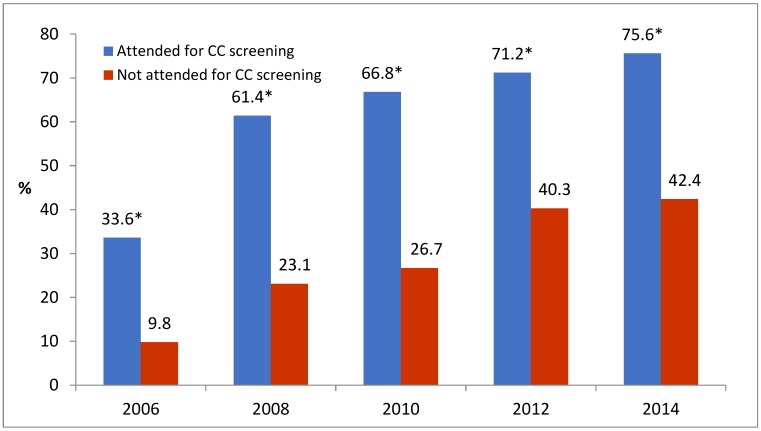
The proportion of women aged 50–60 years having had a mammogram within the last two years, according to attendance for cervical cancer screening in 2006–2014. * *p* < 0.001 compared with nonattenders; CC—cervical cancer.

The attendance for BC screening was associated with the participation in cervical cancer screening (Table 2). During the study period, the proportion of women who reported having had both a mammogram and Pap smear test for cervical cancer increased by almost three times: from 20.1% in 2006 to 58% in 2014. Meanwhile, the proportion of women who did not participate in both screenings decreased from 36.2% in 2006 to 13.4% in 2014. The participation rate in BC screening within the last two years was much higher among women who reported the attendance for cervical cancer screening within the last three years compared with women not being screened for cervical cancer (Figure 2).

Multivariate logistic regression analysis for associations between attendance for BC screening and analyzed factors showed that women with a high level of education were more likely to have a mammogram than those with intermediate or low education (Table 3). The odds of participation in BC screening were 37% higher for women with high education than for those with low education. Living in cities increased the likelihood of having a mammogram almost twice as compared with women living in towns and villages. Marital status and ethnicity were not associated with attendance for BC screening.

The odds of receiving a mammogram increased with the increase in the number of visits to a doctor during the last year. Women who visited a doctor at least three times had approximately 10 times higher odds of participation in BC screening compared to those who reported having no visits.

Unhealthy behaviors decreased the likelihood of attendance for BC screening. Women consuming fresh vegetables less often than daily or exercising during leisure time less than two days a week had between 35% and 24% lower odds of receiving a mammogram, respectively, compared to daily vegetable consumers and those who were more physically active (Table 3). Consumption of strong alcoholic drinks at least once a week reduced the likelihood of participation in BC screening by 34%. No association was found with smoking and attendance for mammography screening.

## 4. Discussion

BC is the most commonly occurring cancer in Lithuanian women. According to the data of the Lithuanian Cancer Register of the National Cancer Institute, the incidence of BC in the age group 50–64 years was 177.1/100,000 [28]. BC screening is intended to increase the detection of early stage cancers and improve survival [11,12]. The coverage of the target population is very important for the effectiveness of BC screening. Our study demonstrated that, in Lithuania, attendance for mammography screening continuously increased from 2006 to 2014. In 2014, 65.8% of women indicated having had a mammogram within the last two years. It is interesting to note that the National Health Insurance Fund reported a lower proportion of women screened for BC (44.9% in 2014); however, the increasing trend in screening attendance was also observed [15]. This difference can be explained in part by coexisting opportunistic BC screening in the country. In addition to the general practitioners, other specialists may refer a woman to mammography. The National Health Insurance Fund does not collect information about the mammography tests outside the screening program. In our study, women were asked about the frequency of any mammography; therefore, our data showed a higher proportion of women having had a mammogram. It is worthwhile noting that Lithuania is one among three countries in the European Union with no central invitation system for screening [15]. This could be one of the reasons for inadequate attendance that does not reach the level recommended in European guidelines—more than 70% [13]. Evidence suggests that introduction of a central invitation system through population register can increase the attendance for BC screening and improve the screening coverage compared to opportunistic screening, which depends on the initiative of an individual and/or a health professional [5,16].

Equity aspects are also very important for successful implementation of BC screening; however, inequalities in attendance still exist in many countries [25,29,30]. Our data are consistent with previous results showing that a higher level of education is associated with the higher rates of participation in BC screening [18,19,20,30]. Better educated women have a greater interest in health, more knowledge about health issues, and better access to resources for health improvement [31]. A study carried out in Switzerland revealed that less-educated women had worse knowledge and more negative attitudes regarding mammography screening compared to women with a higher level of education [30]. On the other hand, education may have an impact on health through work and economic conditions. Low education might be related to lower income. Women from more socioeconomically disadvantaged backgrounds may have a higher proportion of daily life problems, which may reduce their ability to participate in preventive screenings [32]. Paléncia et al. compared different countries in Europe and found that the educational gradient was less pronounced for organized screening [16]. Opportunistic BC screening depends on the frequency of visits to a doctor and the activity of medical personnel in providing information about screening. Women with a higher level of education potentially have more information and greater contact with physicians, and they may, therefore, be more likely to be invited to participate in the screening. Organized screening and screening awareness campaigns targeting less-educated women may help to improve their attitudes and increase attendance.

Our findings on higher rates of having a mammogram among women living in cities compared to villages are in line with other studies [33,34]. However, a study carried out in Korea showed the opposite result: a rural residence was related to higher rates of BC screening attendance [19]. The authors discussed that this result could be partly explained by the mobile screening service provided by the National Cancer Screening Program that increases accessibility and compliance with the screening program [19]. Lower access to mammography screening, distance from health-care services, shorter working hours of health centers, and lower quality of equipment might be barriers to participate in BC screening in rural areas [35,36]. Age and marital status were not associated with participation in mammography screening, consistent with some studies [19,32], but not with other studies [20,24,25,37].

In our study, ethnicity was not associated with attendance for BC screening. The Lithuanian population is quite homogenous. According to the Census 2011 data, 84.2% are Lithuanians, 6.6% Poles, 5.8% Russians, and 3.4% other ethnicities [38]. In Lithuania, access to health care does not depend on ethnicity; however, barriers for screening attendance may differ between ethnic groups. Future studies on this topic are therefore needed.

Our data support previous findings that the number of visits to a doctor is positively associated with the participation in mammography screening [25,39]. In Lithuania, BC screening is more opportunistic than it is organized, so more frequent contact with general practitioners increases the probability of being invited to participate in the screening. Additionally, other studies demonstrated that high comorbidity was associated with better attendance for screening because of more contacts with health-care providers [20,24,39].

The previous studies showed that participating in any cancer screening increases the attendance for other cancer screenings [19,40,41,42]. Our data indicated that women having had a Pap smear test within the last three years were more likely to be screened for BC cancer within the two last years. Over the whole study period, 45.4% of women participated in both screenings. Very similar results were found in a study carried out in Korea, where 51.7% of women ever had both a mammogram and Pap smear test [19]. In France, 32.1% of studied women participated in cervical cancer and BC screening, and 46.2% in screenings for cervical, breast, and colon cancers [41].

Health attitude may have an impact on health behavior. We observed a negative association between some unhealthy behaviors and attendance for BC screening. Women not consuming fresh vegetables daily, being engaged in physical activity less than two times a week, or drinking strong alcohol at least once a week were less likely to be screened for BC. Other studies also confirmed that a low level of physical activity has a negative effect on participation in BC screening [18,20,40]. The data about the effect of smoking and alcohol consumption on BC screening attendance are contradictory. Some authors revealed the negative association [18,42], while others did not find any association [20,32] or reported opposite results that individuals never consuming alcohol and never smoking had a lower likelihood of participation in screening [24].

Analysis of barriers limiting attendance for BC screening may help to develop suitable interventions that improve access to good-quality screening and increase the effect of it. Evidence suggests that inequalities in mammography screening and BC survival could be reduced after the implementation of an organized population-based screening program [16,22,29]. Some strategies were found to increase participation of women from lower socioeconomic groups in BC screening, including cost-reducing interventions, greater involvement of primary health-care specialists, and individually tailored communication that addresses barriers to screening [29]. Recently, Lithuania started to introduce systematic personal invitations for BC screening in some regions.

The strengths of our study include the usage of nationally representative data collected following the same methodology over eight years. The same questions on participation in BC screening were used in all surveys, ensuring comparability of data and the possibility to assess the trends. Furthermore, multiple sociodemographic and health-behavior characteristics of women were collected, enabling us to identify predictors for BC screening attendance.

Several limitations of the study should be mentioned. First, the data were self-reported. Several studies demonstrated that self-reports overestimate the participation rates in cancer screening [43,44]. However, the validity of self-reported mammography data was demonstrated in other studies [45,46]. A meta-analysis of the accuracy of self-reported mammography screening compared to medical-record reported sensitivity of 94.9% and specificity of 61.8% [44]. In a Norwegian study, the overall sensitivity and specificity for self-reported BC screening were 99.9% and 84.4%, respectively [46]. Second, response rates declined across the survey years despite an increase in the level of efforts, such as the use of respondent incentives and information about the study through mass media. Declining response rates and increasing nonresponse rates were observed in many recent national and international studies [47,48]. Multiple reasons, such as the rise of online surveys and increasing survey burden, greater awareness of privacy issues, and altered work/life balance, may affect the intention of the individual to respond. The respondents generally tend to have higher socioeconomic status and report better health and health behavior than non-respondents [49,50]. This may result in an overestimation of participation in BC screening. However, several studies proved that nonresponse does not have any statistically significant effect on associations between variables [50,51]. Next, we were not able to separate the participation in BC screening programs and opportunistic screening. Finally, we did not examine some other factors like cancer family history and psychosocial factors that may have an impact on attendance for BC screening.

## 5. Conclusions

In Lithuania, attendance for BC screening is continuously increasing; however, it is still inadequate. Participation in cervical cancer screening increases the possibility to participate in BC screening. Having a higher level of education, living in the city, making frequent contact with a doctor, and practicing healthy behaviors are significant predictors of attendance for BC screening. These patterns suggest that there is a need to monitor and address the lower uptake of mammography in women with lower education and health-care use, as well as those living in rural areas and having unhealthy behaviors. Our findings provide the evidence that supports the need for an organized population-based BC screening program. Future research is needed to show if a systematic personal invitation system that has been launched in Lithuania will lead to better coverage and less inequality in attendance for BC screening.

## Figures and Tables

**Table 1:** Characteristics of the study population (%).

Characteristic	Study Years				
	2006 (*n* = 345)	2008 (*n* = 363)	2010 (*n* = 451)	2012 (*n* = 393)	2014 (*n* = 386)
Education					
Low	41.4	36.9	35.9	37.2	35.5
Intermediate	36.5	39.7	37.3	38.7	36.3
High	22.0	23.4	26.8	24.2	24.2
Place of residence					
Cities	46.3	53.4	40.1	41.7	46.1
Towns	35.1	30.3	28.2	31.8	32.1
Villages	18.6	16.3	31.7	26.5	21.8
Marital status					
Married	62.3	61.5	65.1	62.1	64.0
Unmarried	37.7	38.5	34.9	37.9	36.0
Ethnicity					
Lithuanian	83.13	85.2	87.4	84.3	88.7
Others	16.9	14.8	12.6	15.7	11.3
Participation in cervical cancer screening *	54.4	60.0	70.1	69.7	71.3
Visits to a doctor during the last year					
No visit	14.3	6.0	8.1	7.6	7.4
1–2 visits	29.9	28.1	28.6	25.5	28.1
3 and more visits	55.8	65.9	63.3	66.9	64.6
Fresh vegetables					
Daily	20.9	26.7	25.1	29.3	32.4
Less often	79.1	73.3	74.9	70.7	67.6
Current daily smoking					
Yes	9.6	10.2	9.3	9.3	9.1
No	90.4	89.8	90.7	90.7	90.9
Strong-alcoholic-drink consumption at least once a week					
Yes	8.5	9.5	8.3	6.1	10.2
No	91.5	90.5	91.7	93.9	89.8
Leisure-time physical activity					
≥2 days/week	52.8	50.1	57.6	61.0	56.6
<2 days/week	47.2	49.9	42.4	39.0	43.4

* Women aged 50–60 years.

**Table 2:** Distribution of the respondents * according to participation in BC and cervical cancer screening in 2006–2014 (%).

Study Years	Participated in both Screenings	Participated only in BC Screening	Participated only in Cervical Cancer Screening	Did not Participate in Both Screenings
2006	20.1	3.9	39.8	36.2
2008	39.0	8.4	24.5	28.1
2010	51.7 **	6.0	25.7	16.6 **
2012	54.1 **	9.7	21.9	14.3 **
2014	58.0 **	9.9	18.7	13.4 **
**Total**	45.4	7.6	25.9	21.1

* Women aged 50–60 years eligible for both screenings; ** *p* < 0.05 compared with 2006 and 2008 years (Z-test with Bonferroni correction).

**Table 3:** Odds ratios of attendance for mammography screening within the last two years, according to sociodemographic and health-behavior factors and visits to a doctor (multivariate logistic regression analysis).

Variable	Attendance for Mammography Screening		
	OR	95% CI	*p*
Age	0.99	0.97–1.02	0.951
Education			
Low	1		
Intermediate	**1.30**	1.01–1.66	0.040
High	**1.37**	1.05–1.78	0.019
Place of residence			
Cities	1		
Towns	**0.52**	0.41–0.66	<0.001
Villages	**0.52**	0.40–0.69	<0.001
Marital status			
Married	1		
Unmarried	0.93	0.75–1.16	0.538
Ethnicity			
Lithuanian	1		
Others	0.92	0.68–1.25	0.604
Visits to a doctor during the last year			
No visit	1		
1–2 visits	**4.91**	2.96–8.14	<0.001
3 and more visits	**10.15**	6.22–16.56	<0.001
Consumption of fresh vegetables			
Daily	**1**		
Less often	**0.65**	0.52–0.82	<0.001
Leisure-time physical activity			
≥2 days/week	1		
<2 days/week	**0.76**	0.61–0.93	0.009
Daily smoking			
No	1		
Yes	0.98	0.68–1.41	0.925
Strong-alcoholic-drinks consumption at least once a week			
No	1		
Yes	**0.66**	0.45–0.97	0.037

Abbreviations: OR—odds ratio; CI—confidence interval. Significant OR values in bold.
